# Friend for the host foe for the guest: Evidence from probiotic *Lactobacillus rhamnosus* GG

**DOI:** 10.1002/imo2.47

**Published:** 2024-12-23

**Authors:** Einar Ringø

**Affiliations:** ^1^ Norwegian College of Fishery Science, Faculty of Bioscience, Fisheries and Economics UiT The Arctic University of Norway Tromsø Norway

## Abstract

The human‐derived probiotic *Lactobacillus rhamnosus* GG (LGG) improves intestinal health in humans. However, in zebrafish, the SpaC pilin of LGG and the LPS produced by SpaC‐induced dysbiotic gut microbiota cause intestinal pyroptosis and epithelial damage. This negative effect is host‐specific, raising concerns about the safety of using classical terrestrial‐derived probiotics strains in aquatic species. These results emphasize the need for species‐specific probiotic evaluations and promote the regulation of non‐host origin probiotics in aquaculture. Further research is needed to understand the action mechanisms of non‐host probiotics and their impacts on economical fish species.

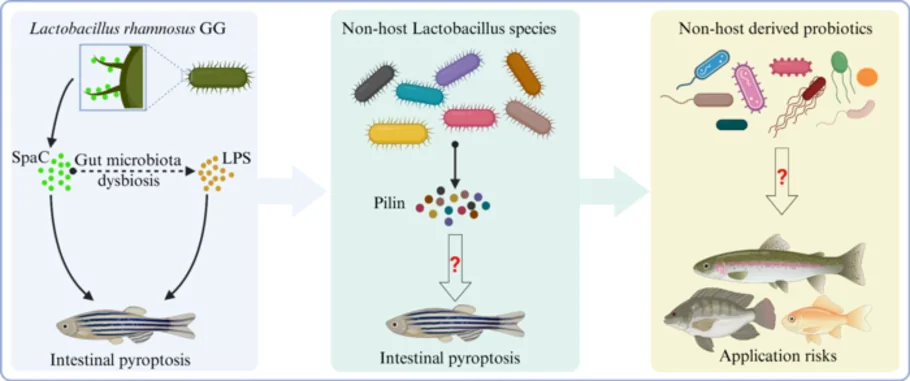

Nowadays, a plethora of documented evidence reveals the significant role of probiotics in improving the growth and overall health of farmed aquatic animals and their environment. The innovation of new omics technologies and computational advances, including microbiomics and bioinformatics, has allowed scientists to gain deeper knowledge about the properties and functions of probiotics, and their mechanisms how affect the host's physiological and metabolic functions. Application of host‐specific probiotics to humans is regulated by the World Health Organization [1], while this is not always for food‐producing animals including aquatic animals. Currently, many probiotics for aquatic animals originate from terrestrial animals as these probiotics have been well‐studied for several decades and have proven effective in promoting growth and health in various terrestrial species. In contrast, the application of probiotics in aquaculture is a relatively newer field, gaining significant attention only in the past few decades [2]. In addition, the intestinal microbiome, as well as the morphology and functions of the intestine of fishes, are different from that of terrestrial animals [3, 4]. Due to these differences, the application of terrestrial‐origin probiotics may not always be as beneficial to aquatic animals. Therefore, the deeper investigations of the application of non‐host origin probiotics and their action mechanisms on the health of aquatic animals are needed.

*Lactobacillus rhamnosus* GG (LGG), originally isolated from fecal samples of a healthy human adult, is one of the most characterized and used probiotics in humans with well‐documented health‐beneficial effects. According to the Web of Science, the first paper on *Lactobacillus rhamnosus* GG (LGG) was published in 1994, and since then, 3.389 papers, of which 629 review papers have been published. The positive effects of LGG on metabolism and immunity have been extensively studied in both human and mammalian models [5]. Specific to fish, a previous study demonstrated that feeding zebrafish with exopolysaccharides from LGG ameliorated hepatic steatosis by regulating the expression of genes related to lipogenesis [6]. However, the administration of LGG in zebrafish has shown adverse effects. A recently published paper in *iMeta* by Zhang et al. [7] is the first and landmark study that demonstrated the negative effect of *SpaC* pilin (a type of adhesion protein subunit of *SpaCBA* pilus) of LGG on the intestinal health and explained the possible mechanisms of how *SpaC* pilin of LGG affects the health of the intestinal of zebrafish, highlighting the negative effects of the application of non‐host origin probiotic LGG on zebrafish intestinal health. *SpaC* protein subunit is one of the subunits of *SpaCBA* pili, which is recognized as a main adhesive factor and found in many species of *Lactobacillus*, including, *Lactobacillus rhamnosus*, *Lactobacillus casei*, and *Lactobacillus plantarum* [8]. The detrimental effect of LGG *SpaC* in zebrafish suggests that host‐specific application of this bacterial strain needs to be carefully assessed when it aims to improve intestinal homeostasis and immune response positively, and infection resistance in different host species.

## 1 DISCOVERY OF THE GUT‐DAMAGING MECHANISMS OF LGG IN ZEBRAFISH

In this landmark study, a mutant strain of LGG (no *SpaCBA* pilus) and the wild‐type LGG (WT LGG) were used to identify the protein responsible for intestinal damage in zebrafish [7]. The results indicated that the zebrafish group immersed in the solution containing WT LGG for 2 weeks showed histopathological intestinal damage and elevation of serum lipopolysaccharide (LPS). Furthermore, after isolation of the three pilin subunits (*SpaA, SpaB* and *SpaC*), the animal model immersed in *SpaC* showed a higher level of serum LPS compared with groups of zebrafish immersed in *SpaA* and *SpaB*.

Another novel finding of the study by Zhang et al. is the discovery of the pyroptosis caused by LGG supplementation [7]. Pyroptosis is a type of programmed cell death that's characterized by its inflammatory nature and is typically triggered by infections or immune responses. The authors further elucidated the Gasdermin (*GSDM*)‐mediated regulatory mechanisms behind this phenomenon using germ‐free and cell models. Gasdermin (*GSDM*) is a family of pore‐forming proteins that can induce pyroptosis after cleavage by caspase. In teleost, Gasdermin E (*GSDME*) is the only pyroptosis‐inducing member of the *GSDM* family and has proven its participation in the response to bacterial infection [9]. The caspase‐3/GSDME pathway is a programmed cell death pathway that can switch between apoptosis and pyroptosis, depending on the expression level of GSDME. The authors observed the caspase3‐GSDMEa pathway was activated in the germ‐free zebrafish group treated with *SpaC*, and illustrated how *SpaC* directly damaged the intestinal mucosa of zebrafish. Cell swelling, shrinking of organelles, rupturing of the cell membrane, and releasing of cytoplasmic contents including pro‐inflammatory cytokines were observed during pyroptosis. Furthermore, toll‐like receptor 4ba (*TLR4ba)* protein was identified as the potential receptor of *SpaC* pili as the expression of this receptor protein was notably upregulated in the intestinal of zebrafish immersed with recombinant *SpaC*. TLR4ba is a protein receptor that plays a key role in the innate immune system by recognizing pathogens and activating immune responses. To verify whether the *TLR4ba* is a receptor for *SpaC* in zebrafish, authors developed the bimolecular fluorescent complex of *SpaC* and the extracellular domain of zebrafish (*zTLR4ba*) in human embryonic kidney 293 cells through bimolecular fluorescence of complementation. This cell model assessment proved for the first time, *TLR4ba* is a receptor protein for *SpaC* in zebrafish and crucial for intestinal pyroptosis induced by *SpaC* and verified that intestinal pyroptosis initiates through the activation *SpaC*‐ *TLR4ba* pathway. The ability of *SpaC* of LGG in activating TLR2, but not TLR4, in mammals' cells, was demonstrated. In humans and terrestrial animals, the activation of TLR2 can lead to the production of beneficial pro‐inflammatory responses, which are crucial in controlling infections and promoting bacterial clearance and enhancing the immune response of the host [10]. The differences in the receptor of *SpaC* protein of the LGG between the fish and mammals may be the reason why LGG is a probiotic for mammals but causes damage to the intestine of zebrafish. Furthermore, the fish TLR4 is paralogous, not orthologous, with human TLR4 [11], and except for some fish species, including grass carp, catfish and zebrafish, TLR4 has been lost from the genomes of other fishes [12]. Related to this a study showed that supplementation of LGG in the diet of Nile tilapia (*Oreochromis niloticus*) which has no TLR4 did not cause intestinal damage as the fish has no TLR4 [13].

This study also highlighted that the fish gut microbiota is affected by the LGG which could serve as one of the mediators to trigger the elevated LPS in the fish. To decipher this, feeding of zebrafish with *SpaC* was conducted, and the results showed that in the zebrafish group fed with *SpaC*, the balance of gut microbiota showed a dysbiosis sign through increasing the abundance of LPS‐producing bacteria such as Enterobacteriaceae. It has been reported that zebrafish inflammatory caspase, *caspy2*, directly senses cytosolic LPS to mediate pyroptosis [14]. Zhang et al. further demonstrated that LPS produced by the dysbiotic microbiota activated the *caspy2*‐*GSDMEb* pathway in germ‐free zebrafish, and this pathway partly accounted for the LGG‐induced intestinal damage. Overall, this study is the first to provide comprehensive and theoretical findings on how the human‐derived LGG could lead to critical health issues in the fish species.

## 2 IMPLICATIONS AND FUTURE PERSPECTIVES

**FIGURE 1:**
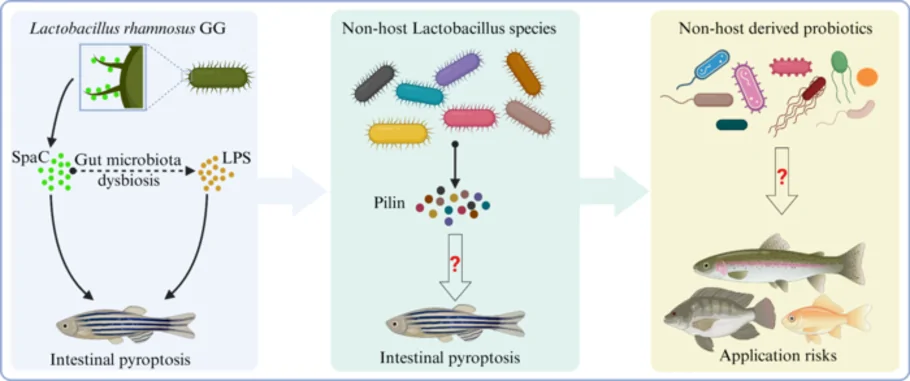
The risk of non‐host origin probiotics on fish. The SpaC pilin of *Lactobacillus rhamnosus* GG and LPS produced by SpaC‐induced dysbiotic gut microbiota cause intestinal pyroptosis. Investigation of the effects of non‐host probiotics, specifically *Lactobacillus* species, on zebrafish and generally assessing if there are any potential risks associated with the application of other non‐host derived probiotics in the health of economically important fish species will need further research. The image was created in BioRender.

The *SpaC* subunit protein of LGG induced intestinal epithelial injury in zebrafish, while it had no negative effect on human cells, and this highlighted the species‐specific negative effects of non‐host origin probiotic LGG. Considering these facts, investigation on the effect of pili, especially *SpaC* pilin isolated from other *Lactobacillus* species on the health of fish is crucial as this pilin is found in many *Lactobacillus* probiotics. Furthermore, many other classical terrestrial‐derived probiotic strains, such as *Bifidobacterium*, *Bacillus*, and *Saccharomyces* are widely used in farmed aquatic animals. Further investigation on other classical *Lactobacillus* species and probiotics is crucial to determine the complete impact of these probiotic species on commercially important fish species (Figure 1). The more we understand this complex host‐probiotics interaction, the more opportunities it presents for innovative interventions and therapies that may greatly improve the beneficial effects of non‐host‐derived probiotics in aquaculture.

To alleviate the possible threats on the application of non‐host derived probiotics, the application of host‐derived probiotics is the better way as such type of probiotic is already adapted to many environmental factors around and inside the host. Nowadays, many fish‐derived microbes have been reported and proposed as probiotics for fish farming, and among them, the hallmark work of the discovery and utilization of *Cetobacterium somerae* is an excellent representative, which is a dominant commensal in the intestine of many fish species and has a significant impact on improving metabolism and enhancing disease resistance of fish [15], and such type of probiotic can mitigate the risks associated with the application of non‐host derived probiotics in aquatic farmed animals. Overall, this work provides new insight into the cautious selection and applications of probiotics in aquaculture and terrestrial animals to alleviate the risks associated with the application of non‐host origin probiotics. Especially those probiotics having pili in animals' health, and the investigation of this friend‐or‐foe relationship with the host will need further study. In addition, applications of non‐host origin probiotics in aquaculture need to be regulated and recommended by the Food and Agriculture Organization of the United Nations/World Health Organization to avoid threats to environmental, aqueous species and human health. Even though screening of probiotics for each economically important aquatic animal can be quite challenging, identifying broad‐spectrum probiotics from aquatic animals that can benefit multiple species with similar feeding habits and environments seems more practical and useful.

## AUTHOR CONTRIBUTIONS

**Einar Ringø**: Writing—review and editing; manuscript approval.

## CONFLICT OF INTEREST STATEMENT

The author declares no conflicts of interest.

## ETHICS STATEMENT

No animals or humans were involved in this study.

## Data Availability

Data sharing is not applicable to this article as no new data were created or analyzed in this study. No new data were created in this commentary article. Supplementary materials (graphical abstract and update materials) may be found in the online DOI or iMeta Science http://www.imeta.science/imetaomics/.
